# Intravascular Pneumocephalus: *A Mimicker of Skull Base Fractures*

**DOI:** 10.5334/jbsr.1795

**Published:** 2019-05-09

**Authors:** Aliaksandr Anisau, Filip Vanhoenacker

**Affiliations:** 1 AZ Sint-Maarten Mechelen, University of Ghent, BE; 2 AZ Sint-Maarten and University (Hospital) Antwerp/Ghent, BE

**Keywords:** retrograde venous embolism, air embolism, pneumocephalus, sinus cavernosus

## Case Study

**Figure 1:**
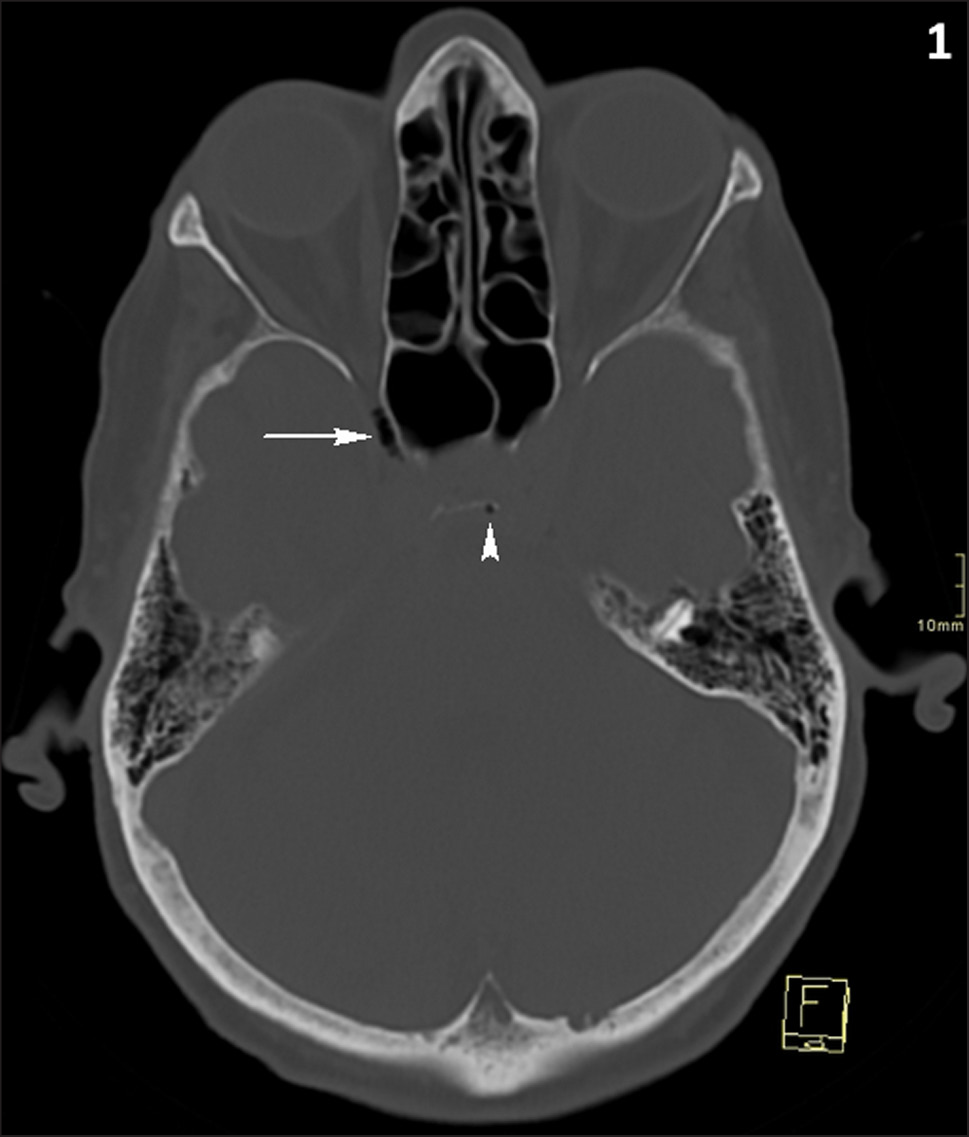
CT of the skull, bone window, axial reformatted image. Air bubbles at sinus cavernosus (white arrow) and sinus intercavernosus (white arrowhead).

**Figure 2:**
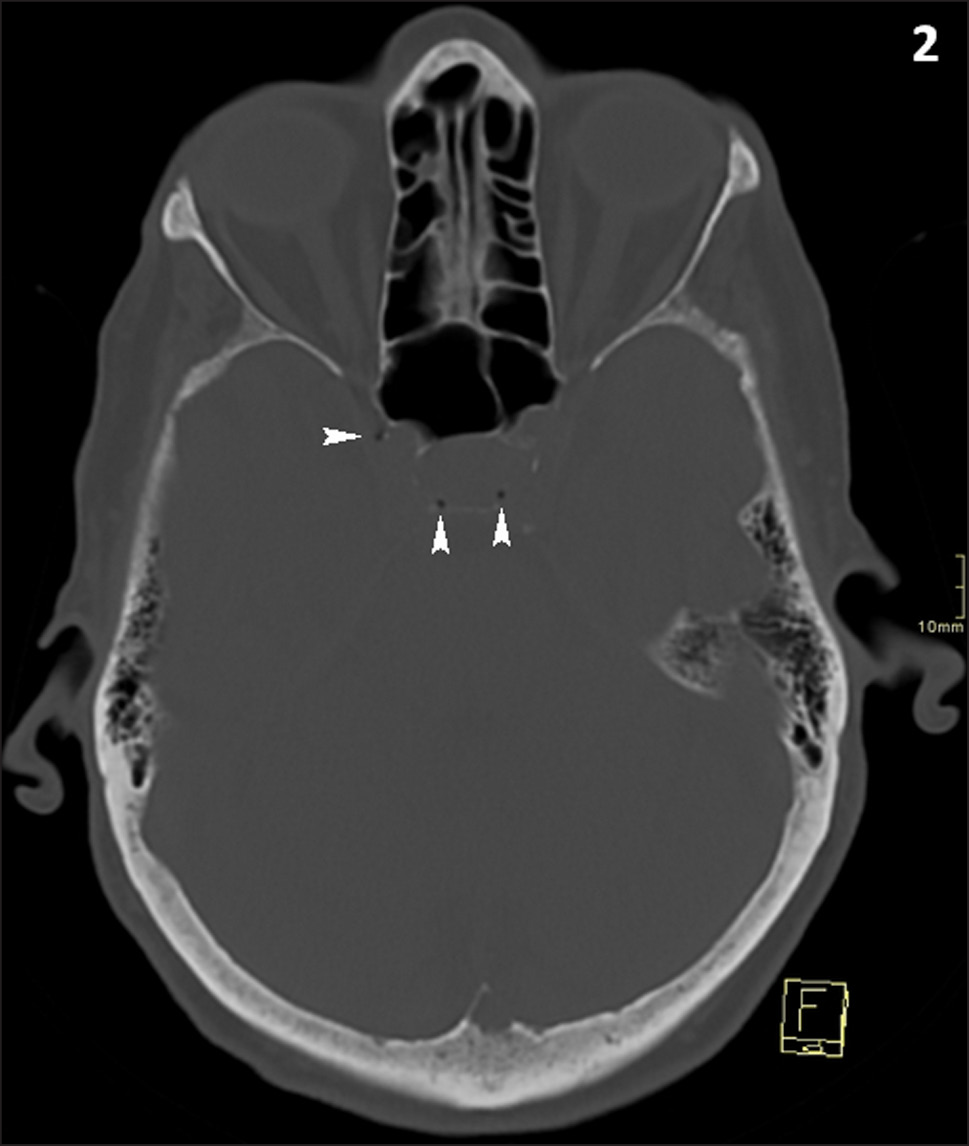
CT of the skull, bone window, axial reformatted image. Air bubbles at sinus intercavernosus (white arrowheads).

**Figure 3:**
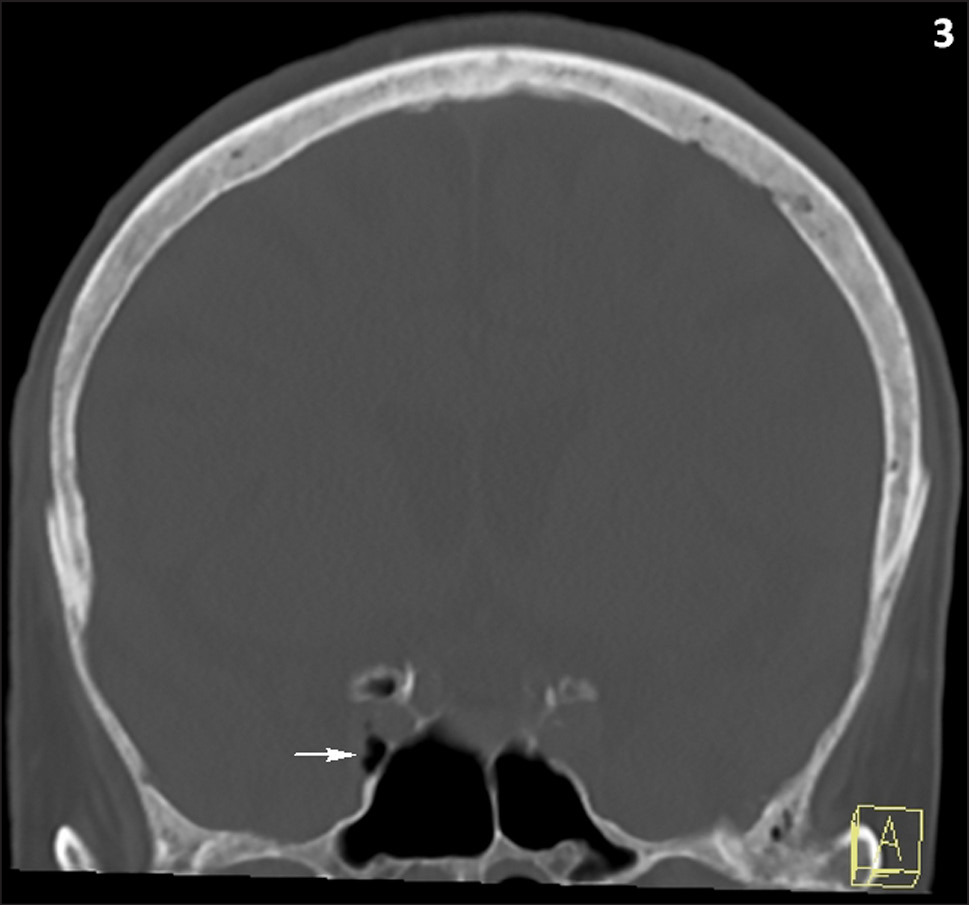
CT of the skull, bone window, coronal reformatted image. Air bubbles at sinus cavernosus (white arrow). Normal pneumatisation of the sphenoid sinuses and absence of a fracture.

A 90-year-old woman underwent computed tomography (CT) of the skull because of head trauma. Approximately 30 minutes preceding the CT, she received a peripheral intravenous infusion with physiological fluid and paracetamol. CT excluded intracranial bleeding, but multiple small intracranial air bubbles were present at the cavernous (Figures 1 and 3) and intercavernous venous sinus (Figures 1 and 2) at the skull base. Although the presence of air bubbles raised suspicion of a skull base fracture, no fracture could be demonstrated and the paranasal sinuses were normally pneumatized.

## Comment

Intravascular pneumocephalus, namely an air embolism into the intracranial vascular system, may be a complication of an intravenous catheterization. It is regarded an uncommon finding, although a prospective study by Thompson et al. exposed six cases out of 100 CT scans in patients with intravenous catheters. Two potential pathophysiologic mechanisms have been described. The air bubbles may spread through a pre-existing anatomical right-left shunt (such as a congenital septum defect or a pulmonary arteriovenous malformation), also known as paradoxal air embolism [1]. Secondly, the air bubbles follow a direct path in the cephalad direction, designated retrograde air embolism [1]. In the latter scenario, the air bubbles get trapped most often in the cavernous sinus. They may also spread to other dural sinuses or in cortical cerebral veins. An upright or semi-seated position of the patient, Valsalva and valve insufficiency of the jugular veins are important contributing factors promoting retrograde venous embolism [1].

An intracranial venous air embolism may be either symptomatic or asymptomatic, depending on localization and extent of the embolism. In symptomatic cases, typical manifestations are mental state alterations, seizures, loss of consciousness, focal neurological deficits, cerebral edema, and eventually death [1]. Cerebral venous infarctions due to gas embolism has also been reported.

Symptomatic cerebral air embolism can be treated by hyperbaric oxygen, no other measures have been proven effective in management of venous air embolism. Avoidance of iatrogenic retrograde cerebral venous embolism is important [1].
